# Reading Between the Lines: A Five-Point Narrative Approach to Online Accounts of Illness

**DOI:** 10.1007/s10912-019-09553-1

**Published:** 2019-04-15

**Authors:** Klay Lamprell, Jeffrey Braithwaite

**Affiliations:** Centre for Healthcare Resilience and Implementation Science, Australian Institute of Health Innovation, Macquarie University, Level 6 | 75 Talavera Road, Sydney, NSW 2109 Australia

**Keywords:** Narrative medicine, Patient-centered care, Medical education, Patient communication, Melanoma

## Abstract

The successful delivery of patient-centered care hinges on clinical affiliation for patients' personal needs and experiences. Narrative competence is a mode of thinking and set of actions that widens the clinical gaze beyond logico-scientific cognition. In this article, we investigate a tool that enables clinicians to rehearse their skills in narrative competence. We apply the narrative competence framework developed by the founding practitioners of narrative medicine to personal accounts of illness and patienthood published on the Internet. We describe our use of the five-point framework in the close reading of 214 accounts by people with the life-threatening skin cancer melanoma.

## Introduction

Training in narrative theory and creative expression—once the domain of those studying the arts—is now recognized as a boon to effective communication in healthcare environments. Medical students and established medical practitioners are learning about story-making (Chretien et al. 2015; Miller et al. 2014) and the role it plays in their relationships with patients and colleagues (Lamprell and Braithwaite 2016, 2017). Through engagement with arts and humanities subjects, they are being given the skills to observe and reflect on the way people convey their experiences and express their needs. These skills, conceptualized as ‘narrative competence,’ help healthcare providers engage in the practice of empathic, collaborative care (Arntfield et al. 2013; Batt-Rawden et al. 2013). Programs that teach narrative competence invite healthcare providers to explore a wide range of narrative forms, from non-medical autobiographical and fictional literature and visual and performance works to case notes, medical charts and taped interviews with patients. The aim is to engage deeply with the people and stories embedded in these works by understanding how “a text’s meaning is carried in the dynamic relationship between what it is about and how it is built” (Charon 2006, 109).

In this article, we investigate a narrative competence framework for reading stories of illness and patienthood published on the Internet. We describe our narrative, interpretive study of 214 personal accounts that have been written by people dealing with melanoma—an aggressive form of skin cancer. These accounts were published on the websites of organizations that support melanoma awareness and research. Unlike serialized blogs and interactive forums, these are ‘short-form’ autobiographical accounts of disease progression and care trajectories. Using a framework developed by the founding practitioners of narrative medicine (Charon 2006; Charon et al. 2016), we explored how narrative competence brings authentic, instructive and reflective engagement with the storytellers of these accounts.

## Background

Recognizing that “medicine has to deal with human personality and human hopes, human fears, and human failings, in conjunction with the material body …” (Clark-Kennedy 1945, 45), physicians are mandated to be patient-centered in their practice of healthcare. Patient-centered care has a narrative imperative—to listen to patients’ stories beyond the bio-medical clues in order to ascertain patients’ personal priorities (Mattingly and Lawlor 2000). In this paradigm, physicians attend to a patient’s telling of his or her “lifeworld” (Mishler 1984) in order to engage in problem-solving care that is personally relevant and sympathetic to the patient (Barber and Moreno-Leguizamon 2017). Alongside the practice of safe, evidence-based medicine, narrative competence is a crucial proficiency in the delivery of gold standard of care.

The rapid development of medical humanities departments in universities and graduate programs in narrative medicine indicates support for a narrative approach to medical pedagogy. As yet, however, there is inconsistent representation of narrative elements in medical programs. Medical education, in the West at least (Huang et al. 2017), is still steeped in logico-scientific thinking (Gupta et al. 2011). Prominence is given to comprehending the bio-medical clues that will lead to evidence-based explanations or which will guide evidence-based investigations. In this mode of thinking, patients’ stories of circumstances “that appear, on the surface, to have little to do with the question” (Riessman 2002, 2) may be perceived as digressions from the aims of a consultation.

The contradictory forces potentially at play between logico-scientific medical training and the ideal of patient-centered care may be intensified by real-world medical practice. Working conditions in which there are stripped-down consultation timeframes, heavy patient loads and demanding administrative and governance requirements make it challenging for physicians to participate meaningfully in patients’ story-telling (Rosti 2017).

In this environment, it becomes important for physicians in training and established practitioners to take up extracurricular opportunities for the development of their narrative expertise. The aim is to practice and rehearse the reflective, imaginative and creative thinking (Viney et al. 2015, 2) needed to “recognize, absorb, interpret, and be moved by” (Charon 2005, 262) the singular experiences of ill health (Oyebode 2010) conveyed in stories.

A foundation method in narrative competence training is close attention to the narrative devices and tools used by storytellers to create their worlds. As Bamberg notes, stories comprise two inextricably linked realms: “the realm of experience, where speakers lay out how they as individuals experience certain events and confer their subjective meaning onto these experiences” and “the realm of narrative means (or devices) that are put to use in order to make (this) sense” (Bamberg 2012b, 3). Attention to the realm of narrative means is a portal to engagement with the realm of experience.

Our interpretive study probed the narrative means used by patients in the stories they publish on the Internet. The accessibility and ever-expanding reach of the Web is making it increasingly possible for people to publish, and audiences to read, a wide range of personal accounts of illness and patienthood. These accounts—also called pathographies or autopathographies (Aronson 2000; Hawkins 1999a)—constitute an emerging sub-genre of narrative expression. We propose that Internet pathographies may be a relevant resource for medical providers seeking to develop narrative competence.

## The study

Our study examined the pathographies of people with the highly malignant skin cancer, melanoma. We chose melanoma because it is a disease that is by anyone at any age, and its incidence is widespread throughout the Western world. Melanoma is an aggressive cancer that forms in the pigment cells of the skin (Melanoma Institute Australia 2017). It can develop in response to UV radiation, but it also occurs in non-exposed parts of the body. Melanoma is categorized clinically in five stages chiefly according to its degree of spread. It begins with Stage 0, a localized occurrence. Stage 1 refers either to the presence of metastases or an ulceration. Stage 2 signifies more advanced disease with the detection of thickening and ulceration. If the disease progresses to Stage 3, the patient now has lymph node involvement. By Stage 4, which is an advanced stage of the disease, there is spread to distant lymph nodes or organs such as the lungs, liver, brain or bone. The incidence of melanoma is rising throughout the Western world (Stamataki et al. 2015) as a result of environmental changes and also as a result of low-cost travel enabling people to travel to UV-high countries (Diaz and Nesbitt 2013).

While advances are being made in chemical treatments for melanoma, particularly in relation to targeted, personalized therapies (Rebecca et al. 2012), melanoma is a deadly cancer. The diagnostic and treatment trajectory of a person with melanoma constitutes a dramatic narrative with logistical and psychosocial implications. People with melanoma face aesthetic outcomes of biopsies and surgeries (Buck et al. 2012; Lee et al. 2016), the functional and psychosocial consequences of side effects from surgery and lymph node removal (Cromwell et al. 2015; Oliveria et al. 2013), and a poor prognosis for those in advanced stages of the disease.

Ongoing surveillance is necessary because melanoma can recur or metastasize many years after a primary melanoma has been removed. People with melanoma thus live with the fear of recurrence (Kasparian et al. 2016), a lifelong dependence on healthcare services and a concern for lifestyle choices that expose them to the sun (Palesh et al. 2014). Melanoma is a complex, unruly disease.

## Method

We spent three months surveying the Internet to understand the environment of patient storytelling specific to melanoma. We identified a relevant and prolific source of patient stories on the websites of organizations that champion research into melanoma and provide support communities for people with melanoma. Many of these organizations offer publishing platforms for people who want to tell their stories. After securing ethical approval, we turned to these websites to establish a ‘population’ of stories for our study.

### Epistemology

In developing an approach to our reading of Internet pathographies, we explored seminal works on narrative form in life stories (Brockmeier 2000; Bruner 1991, 2004; Gergen and Gergen 1986; Genette 1988) and the narration of illness (Kleinman 1988; Mattingly 1998; Mattingly and Garro 2000). We also looked to the formative work undertaken in the interdisciplinary zones between narratology, the humanities and sociology by Arthur Frank (Frank 1995, 2010, 2011) and Anne Hunsaker Hawkins (Hawkins 1999a, 1999b). These authors extensively investigated first person accounts of illness and laid the foundations for understanding illness narratives as a genre of autobiography.

For an explicit methodology by which to attend to the narrative means used in Internet pathographies, we looked to the work of Rita Charon and her colleagues (Charon 2006; Charon et al. 2016). Over the past two decades they have fostered narrative competence as a crucial proficiency for physicians (Charon 2007) and established the field of narrative medicine as a contribution to modern medical pedagogy. Their guidelines for the practice of narrative competence involve the “close reading” (Charon 2006, 116) of texts, attending to the means by which storytellers give narrative form to experience (Bamberg 2012b). They propose, and we implemented, attendance to cardinal narrative features.

### Story collection

The stories we read comprised written, short-form, autobiographical texts describing experiences dealing with melanoma. We sought narrative texts that conveyed the personal impact of those experiences (Garden 2010; O’Brien and Clark 2010). We did not include other formats of Internet pathographies, such as blogs in which the events of a personal experience are serialized over time, because these constitute an unfinished autobiography which is a unique sub-genre worthy of a separate study. Our criteria for inclusion were:

- narrative recounting of a personal experience with melanoma
- autobiographical
- non-fiction
- written
- English language
- published on the Internet
- short-story format, not blog or interactive
- self-generated
- not written for commercial or proprietorial gain

Unsurprisingly, searching the Internet using the terms ‘melanoma illness narratives,’ or ‘melanoma narratives,’ yielded mostly scholarly journal articles. Using obvious synonyms, such as ‘story’ and ‘account,’ yielded a wealth of material; however the content was not necessarily narrative or autobiographical or published in written form. Instead that yield included marketing material featuring patient feedback on healthcare institutions, factual information on melanoma, video and audio compilations of interviews, accounts written by the families and friends of melanoma patients, and contributions to forums. Testing search terms such as ‘personal’ and ‘experience’ yielded more refined results, with a selection comprising 286 short-form autobiographies of melanoma experiences posted to cancer support websites. The final population of 214 melanoma pathographies met the criteria.

### Familiarization with authors

We had limited access to information about the authors of the texts, other than what could be perceived within the texts. Some stories were published anonymously, others were attributed to names that could have been pseudonyms and that did not necessarily indicate gender. Information about the nationality and geographic location of a storyteller at the time of writing was not always made explicit, and given the international nature of the Web, people living in one country were able to publish on the websites of organizations based in other countries. Thus we rarely began a reading of a story with a sense of whether the storyteller was male or female, what age group he or she belonged to or where he or she might live.

It was not always possible either to know much detail about the storyteller’s diagnosis of melanoma. Some websites categorized their stories according to the staging of the disease, so stories were published, for example, under Stage 1, Stage 2, etc. Others published all stories together in order of date of publication with no differentiation related to staging.

Overall, then, what we learned of the ‘profile’ of each storyteller came to us from our close reading of the texts. We were led by mentions of a gender-related experience, or descriptions of conversations with physicians that hinted at the staging of the disease, or mentions of family that hinted at the ages of the storyteller. The stories were read at least once, briefly in the first instance as part of the population selection process, again in the pre-selection process, and thereafter read multiple times.

### Research design and structure

Charon and colleagues propose key prompts by which to attend to the narrative expression of lived or imaginary experience, including time, space, voice, metaphor and genre. As Charon et al. comment, these narrative features may be considered individually, but in practice they work together to produce the unique story that is told—the whole being greater than the sum of the parts (2016, 466). Our comprehension of these narrative features is summarized as follows:

- time—the representation of past, present and future;
- space—the positioning and movement of characters and events within physical and geographical realms;
- voice—the personalities and points of view that are communicated as narrators speak to audiences and characters speak to each other;
- metaphor—the language of resemblance used to describe a feeling, situation or event; and
- genre—the type of text determined by common codes and conventions of narrative construction.

### Procedures

In the initial phase of analysis we established a file for each story using NVivo (v10) qualitative data analysis software. We created personal profiles for each storyteller, categorizing as much demographic, biographic and medical information as could be identified or interpreted. We then coded content according to the a priori codes of time, space, voice, metaphor and genre, beginning with ‘broad sweeps’ of content into each category. We were preparing, as we might in qualitative, paradigmatic studies, to conduct iterative coding of concepts and issues within the categories to arrive at hierarchical thematic sub-categories.

We found, however, that the identification of narrative means resisted such systematic codification. ‘Voice’ for example, featured in every word of every story. Finally our analytic process reverted from paradigmatic to narrative mode of inquiry—we put each story ‘back together’ and used the coding capacity of the data analysis software to highlight and explore the uses of each narrative feature in each text. Two investigators were involved in this project. One read every story and coded the content. Discussions between us evolved the findings.

## Findings

In this section we present examples of our close reading of Internet pathographies. We discuss the insights into, and questions about, the storytellers’ lives that were stirred by engagement with the five key narrative features of the analytic framework we used. This section is presented in a discussion format because the work is interpretive.

### Time

#### Time in illness

In choosing how to represent their lived experiences of melanoma, the storytellers had to establish an event as a beginning point. Half the stories we read began at the point in time when some kind of change in health was identified or a symptom particular to skin disease was noticed. In these accounts we enter the storyteller’s illness experience when the possibility of disease becomes apparent. We move through a timeline in which a diagnosis is made, treatment is given and an outcome is arrived at. Some stories concluded with the storyteller still in treatment, some with treatment finished and follow-up care becoming routine, and others, such as the following story, with recurrence or metastases.

*“At 22yrs I had a small pink nodule on my neck, just behind my ear, my hairdresser commented on it, said it needed to be checked as it went red when she washed my hair. I promised I would have it checked but I didn’t. Over a period of several months it started to turn dark and grow down my neck. Although it wasn’t very big, less than 3mm, people started asking what was on my neck, had I put permanent marker by accident? I finally went off to the doctor and he referred me to a skin specialist and it was removed as a precaution. 10 days later I was told it was a Melanoma and needed further surgery….full check-ups were required for the next 5 years before I finally got the all clear. 28 years later almost to the day, it presented in my gallbladder… I am now stage 4 cancer, living a life of uncertainty.”*

This story spans from early adulthood into middle age though the framing of time is imbalanced. The storyteller is imprecise about time frames in the period of events that lead from symptom to diagnosis, recounting grappling with the presence of the symptom for “several months.” This description notably contrasts with the specificity of periods of time recounted in diagnosis and treatment, follow-up care and metastases—ten days, five years, and twenty-eight years. This contrast prompted reflection about the personal circumstances of the storyteller at the time of symptom identification—what issues untold in the story might have impacted the storyteller’s desire or capacity to take action on so conspicuous a growth for such a long time? The framing of time in the text is also unbalanced by the uncertainty the storyteller speaks of. The short period of the present uncertainty casts a shadow well back into the past—though given the ‘all clear’ almost thirty years prior, it turns out that certainty was never a reality.

The other half of the stories we read began their narrative timelines prior to symptom identification, before recurrence or metastases were identified. In the story extract below, the storyteller’s perception of the experience of illness begins with a time in ordinary life before the disease was exposed. This expositional view frames the experience of melanoma as beginning in childhood:

*“I grew up on a dairy farm... My summer vacations entailed picking rock and stacking bales in the barn. I admit I was young and dumb when it came to skin protection. I rarely used sunscreen. I would go out in the field in a tank top and shorts. I had numerous sunburns. I remember having trouble sleeping some nights due to painful sunburns. There was never a need for a tanning bed because I was always tanned. I was a sophomore in high school when an ugly, irregular, dark mole appeared on my inner thigh. It was bigger than a pencil eraser and appeared to be almost black with a little blue in it. I remember it almost had a numb sensation when it was touched. It was right before my 18th birthday when I noticed the center of the mole had turned white. That wasn't the only change. I was tired all the time! I had also been experiencing a tightness in my throat. It prompted us to visit the doctor. He didn't find anything with my throat, so I was diagnosed with having panic attacks. The doctor looked at the mole and said he didn't think it was cancerous, but he would remove it just to be sure. It was a snowy day in February, a few days before Valentine's Day, when I got the news that I had melanoma…Forty-some stitches later, I was left with a seven inch scar on my leg…I now see a dermatologist annually. I'm 39 years old. I've been married for 16 years and we have three girls. I'm proud to say my daughters have never had a sunburn.”*

This storyteller perceives that she may have lived with melanoma from early on in her life. Though no explicit link is made between the early years of sun exposure and the diagnosis of melanoma, and the storyteller makes no overt comment on how the diagnosis felt in light of the serious sunburns received in youth, she ends the story describing her feeling of pride in preventing her daughters from sunburn. She contrasts the lack of protection from sunburn endured as a child and teen with the present capacity as an adult to protect her own children from that danger. Possible feelings about the early sun exposure and the link to melanoma—regret, anger, guilt—are secreted into the action taken to ensure that the next generation will not have sun exposure to blame for melanoma. Her children will not have to deal with melanoma caused by sunburn in their lifetimes.

Implicit in this story, as in the previous story, is the vague passing of time from symptom appearance to medical advice. The storyteller narrates that she was at sophomore level in high school—which marks an age of between fifteen and sixteen years of age—when the mole appeared and eighteen years old when changes in the mole and other symptoms of ill health prompted the decision to consult a doctor. The appearance of the mole was not enough of a physical change to warrant seeking medical advice. It seems that some two years may have passed from the time the physical change was noted through to the time diagnosis was made and an excision was performed. The storyteller’s self-confessed naivety about skin cancer may have contributed to extending the time in illness.

#### Medical time

We found that many of the Internet pathographies we read were explicit about dates and time frames related to medical trajectories. The following extract offers an example:

*“It started with a black spot on my left shoulder that starting bleeding. On December 31 (new years eve) 2008 the dermatologist called to tell me it was melanoma. Since that time, 2008 to 2013, I have had four surgeries and was told in June 2014 that I was stage 4.”*

While this chronologically organized list of events—this happened then this happened—lacks explicit reference to the physical, psychosocial or practical impacts of the experience, there are implicit clues to the significance of the experience for the storyteller, based on the way time is situated in the story. The storyteller notes, for example, that December 31—the day of the first diagnosis of melanoma—is New Year’s Eve. Though no explanation is given as to why this detail was included or what meaning this date held for the storyteller, we know that the eve of a new year signals renewal and regeneration—a time when it is customary to make positive resolutions about life going forward. We reflected on the impact on our own lives of receiving a diagnosis of cancer at such a time and by empathic inference we could project how diagnosis on this date may have affected the storyteller.

We noted that the stark lack of descriptive detail about events and personal feelings in this story served to illuminate the passing of time. The story ‘slides’ quickly from diagnosis, through four surgeries in five years, to arrive an outcome of another, worse, diagnosis. We wondered if this was the storyteller’s way of emphasizing the role medical treatment had played in that five-year period. We were prompted to consider the storyteller’s experience in the broader context of treatment for melanoma, and to reflect on the limits of surgical intervention in managing this aggressive disease. Four surgeries in five years could not contain the advancement of the melanoma.

Waiting time—the time waiting for test results to be delivered, for the outcomes of surgical procedures to be relayed to the storytellers, for wounds to heal and for melanoma to recur—featured in many of the stories we read. In the following extract, the storyteller frames the impact of a waiting period as a set of uncertainties and questions about his future:

*“The next 21 days were some of the longest; waiting for tests to come back, not knowing exactly how bad the cancer really was, if it had spread, if it hadn’t spread, how long I have to live, what’s next?”*

Another storyteller conveys waiting time as an inflicted period of embargo and disempowerment:

*“After my surgery I would be forced to wait for two weeks before hearing whether or not my lymph nodes were ‘clear’.”*

Waiting has become a prominent aspect of life after melanoma for one storyteller:

*“So this is my life: cutting, stitching, waiting, hoping, fearing and wondering.”*

Medical time was also reflected in the shift from one state of being to another as a result of treatment:

*“I can show you, though, in pictures the difference just a week makes from pre-chemo to one week into chemo. These photos were taken exactly one week apart. I almost didn’t recognize myself in the second photo. The consequence paid for the treatment of this devastating disease was a detachment from the true body and spirit. I can only equate it with a loss of myself. The essence that is Me was gone. I thought at the time that I would never recover that original person that I was. I felt she was gone forever, only to be replaced with a much weaker, feeble version of who I was. My delusion of continuing a near normal life during chemo was long gone…”.*

#### Historical context

Encouraged by new membership of an online community and the increasing ease of publication on the Internet, some storytellers were writing for the first time about events that took place many years ago. Others recounted multiple experiences of melanoma that occurred across decades. Reading individual accounts of historic experiences made us wince in the knowledge that the story might have been very different had the storyteller been diagnosed in a more modern context. The stories exposed times in the past when public awareness of melanoma was limited, when sunscreen lotions were not widely used and when commercial tanning clinics could use high-risk devices.

*“In high school, I would go to the tanning salon occasionally, mostly for prom and other dances. During college, I really liked the way I looked when I was tan, so I started tanning on a regular basis. I went twice a week for about 2 years. I went to a salon that offered a variety of beds. There were beds with 42, 54, and 60 bulbs. I wanted the best tan, so on top of my monthly package, I paid extra for the beds with 60 bulbs.”*

Accounts told also of lack of effective management for side effects of toxic immune suppressant treatments and of surgical procedures that are now irrelevant. In the following extract the storyteller recounts the arduous events of surgery and a skin graft that occurred in 1978, the management of which would be very different today:

*“The operation was in two stages, the first being the removal of a large section of tissue the size of an average saucer and as deep as my shoulder blade, and the removal of a similarly sized patch of skin from the back of my right hamstring, which would later be used to cover the open wound on the back of my shoulder. This piece of skin was removed with an instrument that was virtually like a vegetable peeler. I was kept in an isolation room in a sterile environment as the wound lay open for one week. The doctor had cut a big ring made out of foam and plastered this around the wound with sticking plaster to protect it. After one week the next step was to place the skin from the back of my thigh onto my back. I remember seeing the skin which had been scored with holes so that it could stretch and it reminded my of heavy gauze wire – only yellow in colour. At this point they had to remove the foam and sticking plaster. I remember lying face down on the bed and the doctor saying “this is going to hurt – you’ve got my permission to swear at me!” The pain was unbelievable – I was determined not to swear but there was plenty of really loud “aahhh’s! and Ohhh’s”. I was gritting my teeth so hard I swear I nearly wore them down. Anyway, before long the job was done and my back was in tact again.”*

#### Discussion

Attending to time as a narrative feature of these pathographies allowed us three main ‘points of entry’ into the lives of the storytellers: chronological time as a platform for the unfolding of experiences; the passing of time as a gauge by which storytellers assessed the impact of their experiences; and historic time as a view of the shifting issues individuals have dealt with.

Time also featured as an implicit aspect of the relationship we developed with each storyteller—the time we spent reading and re-reading a pathography and then the time spent thinking about that storyteller long after our study was competed. Our reading of these pathographies visited storytellers at a particular time in their lives, and we were keenly aware that the circumstances of their health and their lives overall may have shifted significantly since the time of writing. A question lingers: what happened to that person? Unlike the experience of reading serialized blogs, a reader of short-form Internet pathographies is given a snapshot into a person’s life and point of view at a particular time, and then left to wonder. The certainty that things have changed since the story was told forms a “continuity/change dilemma” (Bamberg 2012a): we must attend to the story at hand and become absorbed in the life that is being told and yet attend with equal commitment and imagination to the changes that may have occurred in the time since the story was written.

### Space

Storytellers rarely described in detail the physical spaces and geographical places in which events occurred. Rather, these spaces were made meaningful by the intensity and dynamic of the experiences being recounted. For one young storyteller, exiting her parent’s car to see her doctor represented a departure from life as she once knew it:

*“A few weeks later I went back to get the stitches removed. My dad waited with my younger brother in the car and I entered the doctor’s office on my own, knowing it would only take a few minutes. Within minutes, I was rushed into the nearby surgery room after being told I had a stage II melanoma. Twenty stitches later, I returned to the car overwhelmed by my experience.”*

The ordinary spaces that we occupy in day-to-day life offered a context by which storytellers compared the ‘before and after’ impact of treatment:

*“It is always the small things that make the biggest impression. I was leaving our local market after doing the weekly food shopping when I got a flashback of my first attempt at picking up a few groceries by myself after chemo. I was still very very weak and didn’t know if I could do it without my husband with me. He came with me everywhere because of my fear of collapsing due to weakness. I did achieve this task, albeit very slowly. As I remembered this achievement, I remarked to myself what a difference a couple of months made. I was walking to my car with a full basket of groceries at a very quick pace, without any fatigue or weakness at all.”*

*“After my surgeries on my leg, I wasn’t allowed to go to the gym, climb stairs, or even drive my standard car.”*

Physical realms were represented also as relational spaces in which meaningful interactions took place. The relationship between storyteller and physician, for example, is illustrated in the following story via the description of movement—and also stillness—of people within settings:

*“[The doctor] came to my hospital room and asked if I wanted a second opinion. I was so touched that she would sit at my bedside and ask that question…She told me she was going to a big oncology conference and would have more information for me when she got back. A few weeks went by and when she came back from the conference I had an appointment to see her. When I got into the office, I was very excited to see her because she had become part of my life, and I wanted to see what she learned at the oncology conference. I noticed that she [the doctor] had some difficulty looking at me, and I made it easier for her. I said, ‘there isn’t anything new out there for me, is there?’ She said, ‘no, and we just sat there for awhile.”*

#### The body as a space

All storytellers described some aspect of alteration in the physical spaces of their bodies. Commonly, though in unique ways, the body was depicted as the setting for a series of dramatic changes wrought by the disease and by treatment:

*“Many surface skin lesions develop……. basal cell carcinomas, squamous cell carcinomas, melanoma….. Then metastasizing internally……”*

*“The doctors decided to remove my right jaw, that was horrible. I lost my teeth, use of my tongue and all the feeling in my face.”*

Many storytellers described a sense of powerlessness over their bodies. We read in one account how a storyteller’s perceived competence as keeper of her physical realm was diminished by the unruly nature of her changing body. Before becoming aware that she had melanoma, she sought and responded to causal explanations for the changes to her body, enacting a sense of control that soon slipped away:

*“My first blue lump appeared on my wrist in early September 2008. My GP referred me on to a physiotherapist, believing it to be some sort of repetitive strain injury. The physio set some exercises and I dutifully completed them all. In October, I went on holiday believing that all was well. Within days, I started to get pain in my underarms and around my breasts but I put it down to muscular strain as I had been swimming every day after weeks of inactivity I also noted how tired and irritable I was but these I attributed to menopause, my full time work load, my part time study and my two sons. So I was not unduly concerned until the appearance of blue lump number 2. I grew a lump per day and returned [home] for a biopsy…He doesn’t mess about; it’s stage IV melanoma with metastases in my brain, lungs and lymph nodes located under my arms. The blue lumps that appeared on my body now have a name - they’re secondaries but they can’t find a primary site….I know I can’t go back to life as it was, I know I have to take this news on board and go forward. A wise friend uses the word “transcend.” She says I need to enfold this into my previous experience and transcend but I don’t know how to enfold.”*

Storytellers also described the ways in which the once private spaces of their bodies became subject to public inspection and management. The following story excerpt portrays the sense of exposure felt by one storyteller as investigations continued on a mole that had become dark and itchy:

*“The next two months were filled with doctor consultations, blood tests, radioactive dye injections, and the most embarrassing appointment of my life. I would be asked to stand completely naked in a doctor’s office while every inch of my body was photographed for documentation purposes.”*

Surgery was often portrayed as having a significant impact on the relationship between conscious and physical self, especially when storytellers were unprepared for the extent of the excision or the ongoing consequences of surgery on their bodies. The expression of surprise and shock took the form of explicit commentary in the following story:

*“The next afternoon when I was finally somewhat aware of what was going on and in TREMENDOUS pain, the nurses came into change my bandage. I told them that I wanted to see what had happened. They were reluctant to do so, but I was persistent. What I saw next I WILL NEVER FORGET. It was devastating. They had removed over 60% of my ear, they had an 8″ incision curving around my cheek, down to my chest, and a big hole in the side of my head where they removed my ear. It made me nausea and weak looking at it. I had not expected that at all.”*

Yet in another pathography the storyteller expressed disengaged attention to the impact of surgery, portraying his interest in what had happened to his own body as un-natural, and using clinical vocabulary to describe his perception of the surgical wound:

*“I had the surgery, Wide Local Excision and was surprised by the amount of tissue they removed. Morbidly enough I took a picture of the specimen and stitches before they packed me up. They cut down to the muscle.”*

Expressions of the body as a space imposed upon by disease and by treatment were inextricably linked with descriptions of psychosocial and existential states of being. One storyteller declared her body as a meaningful site by tattooing her leg with an inscription:

*“Almost one year to the day of my surgery I got the melanoma ribbon tattooed my leg with the quote ‘one day, one breath, one step at a time.’ It reminds me daily of just how strong I truly am!”*

#### Discussion

In attending to space as a narrative feature, we became aware of the ways in which physical realms and geographic entities shaped storytellers’ recall of their experiences. Simple references to locations such as clinics and car parks became meaningful as storytellers described the personal significance of the events that occurred in those spaces. Our engagement with storytellers was perhaps most highly drawn by their portrayals of bodies as physical spaces undergoing transformation. Given the aggressive and recurrent nature of melanoma, storytellers’ accounts often told not only of significant aesthetic and functional consequences of melanoma and melanoma treatment but of the ways in which the changing spaces of their bodies shifted their fundamental perceptions and beliefs.

### Voice

#### Vocal cues

By attending closely to the linguistic components of the pathographies, we developed an awareness of how storytellers composed their texts to reflect a particular voice. Punctuation was a foundational tool for storytellers who wanted to create a sense of fervor in the text. Intensity of feeling was indicated by extensive use of exclamation marks, question marks and capital letters, extended ellipses and typographical symbols to indicate swearing.

*“I couldn’t believe it. Cancer?? Me??? How is this possible? I had a 2 year old and a newborn! What a cruel thing to have this happen after bringing such sweet lives into this world.*”

*“……with shock having taken over, I blurted out, “ARE YOU ##%%! KIDDING ME?” The thought of my eye ball being cut and frozen and…………………..It’s all just too much. I break. I break down….right there, with this nice eye tumor specialist and his nurse, I just sob.”*

*“Prior to my diagnosis I had no idea of how aggressive and horrible melanoma is. I thought, ‘Skin cancer – that’s no big deal!’ Boy – was I WRONG!!!*”

Some storytellers repeated letters to enact the written version of what would have been their spoken tone. As an example, the following storyteller emphasized the rationalization of his or her distress by lengthening the word ‘oh’ in the phrase ‘oh well’:

*“Sleeping at night is hard too between the anxiety and my leg falling asleep no matter what position I'm in. But ohhh well, I always think it could be worse.”*

#### Point of view

The pathographies we read, with one exception, used a first person point of view. As readers responding to the conventional “autobiographical pact” (Lejeune et al. 1977) of the first person, we understood that the ‘I’ and ‘me’ of the story was the author narrating a story about him or herself to us. Some storytellers emphasized this relationship by using the second person in speaking to us, the readers:

*“This whole scenario was like a nightmare, as most of you have probably experienced the same thing.”*

The choice by one divergent storyteller to use the third person point of view highlighted the complex relational act we engaged with in reading these pathographies.

“*All it took was one freckle she happened to spot on her back - the size of a tip of a pencil. It doubled in size, then tripled. Yet, still unnoticeable to most around her. But the warnings of her mother's irreversible scars rang through her ears. Then were spoken aloud by her doctor. Then were coming true as the specialist took wide enough margins of her flesh to ensure the freckle - that pesky little freckle - would be completely excised. The size of a tip of a pencil turned into stitches on her scapula, where the skin is constantly moving, shifting, expanding, contracting. The stitches turned into a deep enough scar to fit a pinky finger. The size of a tip of a pencil. That's all it took.”*

Why had this person decided on the use of the third person in the telling of her lived experience? We wondered if the intention was to distance herself from the experience, to engage our attention by being different, or perhaps to bring her experience into a literary domain.

#### Through the voices of others

Storytellers also drew on representations of conversations with others to establish their voices in the texts. Using prose descriptions and verbatim portrayals, storytellers displayed their personal traits in interactions with family members and friends, physicians and other healthcare professionals, non-healthcare professionals such as hairdressers and beauticians, and other melanoma patients introduced to the storytellers via physical and online patient support groups:

*“I laughed nervously and responded, ‘But it's not cancer cancer?’ He looked me in the eye and stated that there is only one definition of cancer and that skin is the largest organ of the body. I felt like I was drowning in fear and panic. I had no health insurance. I needed to call my parents. Finally, I stuttered the question, ‘Could I die?’ And so my journey began.”*

*“My surgeon, bear with me, I get great pleasure out of calling him ‘MY’ surgeon. He was very serious when he moved me to the top of his surgery list and as I handed him back the signed forms he noticed my little note saying ‘while you’re in there, can I have a C cup? Just joking’ I laughed, ‘not really’ I whispered. He told me he would give me a pretty scar. He is the perfect perfectionist. 18cm long shark bite, you can hardly notice it now.”*

#### Discussion

Using the narrative feature of voice as a prompt for delving into each text, we came to know the tone, style and personality of the storytellers as narrators. Few storytellers were explicit about the challenges inherent in finding a public voice with which to relay private experience. It was easy to assume that the majority felt comfortable and competent in expressing themselves through writing and sharing their stories with others, and in using the medium of the Internet as a publishing platform. Yet as Frank notes, people with serious illness may experience a narrative chaos (1998, 1995). In considering voice as a distinct aspect of the pathographies we read, we became aware of the complex task that was performed by each of the storytellers in making their experiences of melanoma ‘tellable.’

### Metaphor

#### Metaphors for psychosocial states

Metaphorical expression helped storytellers to express the philosophical, emotional and psychosocial impact of the disease and treatment on their lives. The following storyteller likens the feeling of diagnosis to the lack of control and wreckage experienced as a victim of a road accident and likens involuntary immobility to the devastation he or she feels:

*“Well, I felt as though I had been hit by a truck….the reality that this was happening to me was paralyzing….nothing can really prepare you to hear those words. There seemed to be no end to the bad news.”*

The use of the conventional metaphor of being hit by a truck contrasts with the use of original metaphor in the following pathographies. This storyteller also uses vehicles as metaphors by which to measure the impact of treatment:

*“I just finished my third round of chemo yesterday and I’m feeling OK- about 50% capacity. Used to be a V8 super car now I’m a wimpy 4 cylinder running on diesel.”*

Another storyteller combined figurative and symbolic language with metaphor to convey the shifting nature of the relationship between self and disease:

*“My mortality lay down on me like a heavy, itchy, woollen blanket. Every night, it was my loyal companion. Most friends fell away, but the real ones stuck around. My new friends keeping me company were fever, vomiting, and diarrhea. Miraculously, they brought wisdom, insight, and compassion. Day after day, night after night, my real self, my soul, began to emerge. I watched my hair fall out and saw courage, character, and strength grow in. Gone was the self-absorbed beach bunny, and in her place was someone new, but familiar.”*

#### Militaristic metaphors

The use of militaristic metaphors to describe dealing with melanoma was widespread in the pathographies we read, reflecting the culturally paradigmatic framing of cancer in Western cancer discourse as a hero’s journey. Employing metaphors of war, fighting and heroism, many storytellers depicted their relationship with melanoma as adversarial:

*“Coming out on the other end of my battle with cancer I am forever grateful for the friends and family that were there throughout my entire journey.”*

*“I know I am in for one heck of a fight but am NOT going to accept the prognosis and feeling like a death sentence that my wife and I was handed*.*”*

The metaphors of enmity used to frame descriptions of disease and treatment led to the characterization of melanoma as an antagonistic figure:

*“I lost most of my nose to the BEAST.”*

*“I live my life with this ever-present invisible stranger with nothing but bad intentions.”*

*“My life changed forever when an unwelcome intruder came knocking at my door.”*

The combat metaphor also served to position other people with melanoma as fellow fighters, as in the following story:

*“I now want to share my experiences with others and give hope to other melanoma warriors.”*

Descriptions of scars from excisions were gathered into the war metaphor, with some storytellers describing the effects of surgery as ‘collateral damage’ and ‘battle scars’:

*“So, at the age of 31 I have had 4 surgeries, and I have scars on my forehead, my back, my chest, my thigh, and my hip. But every day I look at these scars, I realize that these are my ‘battle scars’, and that I am a very lucky girl.”*

#### Journey metaphors

The metaphor of journey was used commonly to depict the medical progression from diagnosis to outcome, or to convey the shifts in personal qualities and movement in personal relationships that had occurred from the point of diagnosis:

*“I have just reached a major milestone in my cancer journey, 3 years cancer free.”*

*“You tread a personal journey and you carry your cross, and there is only one person who can carry that cross for the duration of that journey. Everyone else is simply lining either side of the road wishing you well.”*

One storyteller considered whether the journey metaphor is an apt means of conveying the experience of melanoma:

*“It seems that every second or third person has a cancer story. A journey, a lot of people call it, and I suppose in a way it is. But my cynical side says “Really!!??” A journey for me would be a little more fun with a dash of adventure. However, to be truthful as with a journey I did discover much about: myself, my husband, my family and friends, acquaintances, the medical system, and my fellow journey mates.”*

#### Discussion

Through our focus on the use of metaphors, we witnessed the use of figurative and symbolic language to describe and explain thoughts and feelings. We noted that some storytellers developed original metaphors while others employed metaphors that are universally shared. We observed the ways in which these metaphors shaped storytellers’ personal and communal identities. Metaphors appropriated from the identity narratives of the wider cancer community, for example, positioned storytellers as selves at war with melanoma. We noted also that the kind of metaphor used was a mediating factor in our level of engagement: the more familiar the metaphor, the more quickly we understood the storyteller’s meaning, though it was also true that metaphors which were original and highly personal demanded greater consideration and compelled a deeper engagement with the text.

### Genre

In our readings of the 214 stories we became aware of a pattern in the construction of these texts. The descriptive narrative of events—the ‘what happened’ of the story—was structured to unfold in chronological order leading from symptom recognition or diagnosis through to outcome, but was often ‘topped and tailed’ with personal commentary. These introductory and concluding aspects of the texts can be conceived in literary terms as prologue and epilogue. Using these structural devices, the storytellers introduced themselves to their audiences, and expressed their overall thoughts and feelings about their experiences of melanoma and melanoma treatment.

#### Prologues

Just over half the storytellers (51%) used a prologue to familiarize readers with the personal nature of the story that was about to be recounted. As an example, the following storyteller expresses the emotions that are involved in telling his or her story:

*“As I sit down to write this, my body is already going into fight mode. I am clammy, scared, and have a stomach full of knots. Why? Because my story is real!”*

Prologues were a means by which storytellers could herald the wisdom they had gained from the story they were about to tell and promote causes for which they had become advocates:

*“In 2011 my life as I knew it ended for good as I was diagnosed with stage 3 Melanoma. Yep, you read it right, cancer at 25 years old! Cancer doesn't discriminate because of age, gender, race, looks or popularity, it picks anyone it likes. Little did I know that what I was about to through would be more challenging than I can ever describe to you. However, I would fight and I would survive and I can now say I am 10 times the person I ever could have been without battling cancer, for me it was a blessing in disguise.”*

*“Hi, I decided to write about my story, hoping that other young and old girls will stop going to tanning beds!”*

#### Epilogues

Most (78%) storytellers used an epilogue to complete their pathography. In their epilogues, storytellers summarized what had been recounted and reflected on the personal significance of the experience of melanoma.

For many, that significance was to be found in the personal transformations that had occurred or continued to occur as the experience continues:

*“I spent so much time crying and feeling sorry for myself that now I just get angry and determined to get healthy, get rid of this, and live the life my family and I deserve.”*

Overwhelmingly, personal transformation was depicted as positive and the challenges of melanoma were framed as a ‘hero journey’ in which existential boons were acquired along the medical trajectory:

*“My life was very fulfilling prior to cancer, but cancer has given my life a degree of fulfillment that I never thought possible. Cancer is not a death sentence. It is only as powerful as you allow it to be. I personally have taken more from cancer than it will ever take from me, and that, my friends, is a win.”*

The narration in these epilogues often took a tone of advocacy, bestowing insights gained from the experience with melanoma on readers and promoting an ideal perspective:

*“Life really is short, it should be treasured on a daily basis. Will it be a truck, cancer, old age, armed robbery, collapsed mine? No—one knows it really is out of our control. Take the days and nights given, strive to love and appreciate it for what it is worth.”*

In contrast, epilogues also provided storytellers with platforms for expressing the ongoing chaos and fear wrought by melanoma on their lives:

*“I try to be positive but all I can think about is my 7 week old baby. I'm terrified to find another spot, I'm terrified to go outside. I'm honestly just a mess right now.”*

Epilogues also provided storytellers with a means of projecting from their current state into the possibilities of the future. For some this was a fearful proposition, as this storyteller conveys:

*“I am afraid of my own skin and I don't wish that upon anyone. I've been told that getting melanoma multiple times is rare and I guess I am one of those rare cases. I have also been told that I am a ‘high risk’ patient and that I will have to deal with this the rest of my life.”*

#### Discussion

Prologues and epilogues featured repeatedly in the Internet pathographies we read, establishing these structural devices as conventions of the genre. By bookending their chronicles of events with commentary, storytellers would lead us from their present thoughts and feelings into their past circumstances and then bring us back into the present or take us into their projected future. The persona of storyteller in a prologue and epilogue was that of the current ‘self’—the self who was writing—contrasted with the self that existed and evolved in the unfolding events of the narrative. In developing an awareness of these conventions, we became attuned to the way that storytellers might recount their experiences, and sensitized to the scope of perspectives storytellers might offer on their experiences.

## Overarching discussion

We conducted a close reading of 214 web-based autobiographical patient stories. Our goal was to profile narrative competence through engagement with both form and content of online narratives of people with melanoma. We investigated the kinds of insights into patients’ personal experiences that might be gleaned using a framework of five narrative features as interpretive prompts—time, space, voice, metaphor and genre. To our knowledge, this framework has not previously been applied to patient stories published on the Internet.

## Contribution to narrative competence

The findings of our close reading suggest that this framework offers three key outcomes in relation to the development of narrative competence. First, the use of a well-defined framework enables an understanding of the narrative choices patients may make in portraying their healthcare trajectories and expressing the personal consequences and psycho-social impact of the events of their illness and treatment. Second, the framework facilitates analytic, affinitive comprehension of unique and shared experiences of melanoma symptoms, disease treatment and survivorship. And third, by applying the framework when reading patient narratives, or any kind of patient-related text—in formats as varied as clinical notes, specialist referrals and case studies—clinicians will be practicing authentic engagement with the ‘patient as person.’

The outcomes of our analysis highlight the ways in which a five-point narrative competence framework widens the clinical gaze. Taking time, space, voice, metaphor and genre into account when thinking about a patient’s story navigates healthcare providers directly into the patient-centred point of view that enriches the quality, safety and relevance of clinical assessment and overall care.

## Limitations

In our presentation of our findings, we chose to discuss each a priori narrative feature, using examples from texts. We might also have chosen to focus on a selection of whole pathographies, investigating the function of each narrative feature within a single text. This may have yielded different kinds of insights about the storytellers and their experiences.

We may also have attended to the influence of online communities on the choices of content, narrative structure and language made by storytellers. We saw, for example, how the use of some metaphors linked storytellers to wider personal, social and political narratives of cancer, promoting messages of adversarial perceptions of disease and heroic transformation. If cultural narratives inform storytelling, so too do ‘organizational narratives’ (O'Brien and Clark 2012). The narrative choices made by these storytellers may be influenced to greater and lesser degrees by the organizations that sponsor the websites on which the stories are published. Following guidelines and suggestions for content established on these sites and reading other stories published on these sites may shape the choices made by these storytellers.

Though the aim of narrative competence is to affiliate with the experience of individuals, our reading of multiple accounts offered the opportunity to consider and systematically investigate commonalities and patterns in the conventions of Internet pathographies. Had our study been conducted in the paradigmatic (Bruner 1986, 12) mode of narrative inquiry, we might have systematically investigated the pathographies for thematic, probabilistic findings on the use of narrative devices in web-based texts of people with melanoma. Rather our study was a “narrative mode” of narrative inquiry (Bruner 1986, 13)—an interpretive examination of the practices of narrative representation in individual Internet pathographies of melanoma. We drew on narrative theory as a means of gleaning, understanding and explaining aspects of singular lived experience that was not necessarily explicit in the verbatim discourse (Lopez and Willis 2004). Thus our focus was on the way personal choices in narrative representation illuminated the experience of the individual.

## Conclusion

Our broad intention was to honor the efforts of people with melanoma who have told their stories in public forums by attending to their accounts of self and their descriptions of their circumstances. The power of a narrative process to articulate and share personal experiences becomes identifiable in this study’s close reading of melanoma illness stories. Through their structural and linguistic choices in story-telling (genre), their framing of events in past and current contexts and imagined futures (time), their presentation of places and bodies as sites of meaningful transaction (space), their establishment of narrative tone, style and perspective (voice), and their varied approaches to using symbolic and figurative language (metaphor), these storytellers invite our affiliation with their experiences. With the practiced competence to attend to such narrative features, patients’ stories become a resource for patient-centered care.

## References

[CR1] Arntfield SL, Slesar K, Dickson J, Charon R (2013). Narrative Medicine as a Means of Training Medical Students toward Residency Competencies. Patient Education and Counseling.

[CR2] Aronson JK (2000). Autopathography: The Patient's Tale. The BMJ.

[CR3] Bamberg, Michael. 2012a. “Identity and Narration.” In *The Living Handbook of Narratology*, edited by Peter Hühn, Jan C. Meister, John Pier, and Wolf Schmid, http://www.lhn.uni-hamburg.de/article/identity-and-narration [3]. Hamburg University.

[CR4] ———. 2012b. “Narrative Analysis.” In *APA Handbook of Research Methods in Psychology Vol. 2*, edited by Harris M. Cooper, Paul M. Camic, Debra L. Long, Abigail T. Panter, David Rindskopf, and Kenneth J. Sher, 77-94. Washington, DC, US: American Psychological Association.

[CR5] Barber S, Moreno-Leguizamon CJ (2017). Can Narrative Medicine Education Contribute to the Delivery of Compassionate Care? A Review of the Literature. Medical Humanities.

[CR6] Batt-Rawden SA, Chisolm MS, Anton B, Flickinger TE (2013). Teaching Empathy to Medical Students: An Updated, Systematic Review. Academic Medicine.

[CR7] Brockmeier, Jens. 2000. “Autobiographical Time.” *Narrative Inquiry*. 10.1075/ni.10.1.03bro.

[CR8] Bruner, Jerome. 1986. *Actual Minds, Possible Worlds*. The Jerusalem-Harvard Lectures Book 1. Cambridge, Mass.: Harvard University Press.

[CR9] Bruner Jerome (1991). The Narrative Construction of Reality. Critical Inquiry.

[CR10] ———. 2004. “Life as Narrative.” *Social Research* 71 (3): 691-710.

[CR11] Buck Donald, Rawlani Vinay, Wayne Jeffrey, Dumanian Gregory A, Mustoe Thomas A, Fine Neil A, Galiano Robert, Kim John YS (2012). Cosmetic outcomes following head and neck melanoma reconstruction: The patient's perspective. Canadian Journal of Plastic Surgery.

[CR12] Charon R (2005). Narrative Medicine: Attention, Representation, Affiliation. Narrative.

[CR13] ———. 2006. *Narrative Medicine: Honoring the Stories of Illness*. New York, US: Oxford University Press.

[CR14] ———. 2007. “What to do with Stories: The Sciences of Narrative Medicine.” *Canadian Family Physician* 53 (8): 1265–1267. PMC1949238 17872831

[CR15] Charon R, DasGupta S, Hermann N, Marcus ER, Irvine C, Colsn ER, Spencer D, Spiegel M (2016). *The Principles and Practice of Narrative Medicine*.

[CR16] Chretien KC, Swenson R, Yoon B, Julian R, Keenan J, Croffoot J, Kheirbek R (2015). Tell Me Your Story: A Pilot Narrative Medicine Curriculum during the Medicine Clerkship. Journal of General Internal Medicine.

[CR17] Clark-Kennedy AE (1945). *The Art of Medicine in Relation to the Progress of Thought*.

[CR18] Cromwell K.D., Chiang Y.J., Armer J., Heppner P.P., Mungovan K., Ross M.I., Gershenwald J.E., Lee J.E., Royal R.E., Lucci A., Cormier J.N. (2015). Is surviving enough? Coping and impact on activities of daily living among melanoma patients with lymphoedema. European Journal of Cancer Care.

[CR19] Diaz James H., Nesbitt Lee T. (2013). Sun Exposure Behavior and Protection: Recommendations for Travelers. Journal of Travel Medicine.

[CR20] Frank AW (1995). *The Wounded Storyteller: Body, Illness and Ethics*.

[CR21] Frank Arthur W. (1998). Just listening: Narrative and deep illness. Families, Systems, & Health.

[CR22] ———. 2010. *Letting Stories Breathe: A Socio-Narratology*. Chicago, Illinois, US: University of Chicago Press.

[CR23] ———. 2011. “Practicing Dialogical Narrative Analysis.” In *Varieties of Narrative Analysis*, edited by J. A. Holstein, and J. F. Gubrium, 33-52. SAGE Publications.

[CR24] Garden R (2010). Telling Stories about Illness and Disability: The Limits and Lessons of Narrative. Perspectives in Biology and Medicine.

[CR25] Genette G (1988). *Narrative Discourse Revisited*. Ithaca.

[CR26] Gergen KJ, Gergen MMM, Sarbin TR (1986). Narrative Form and the Construction of Psychological Science. *Narrative Psychology: The Storied Nature of Human Conduct*.

[CR27] Gupta Richa, Singh Satendra, Kotru Mrinalini (2011). Reaching people through medical humanities: An initiative. Journal of Educational Evaluation for Health Professions.

[CR28] Hawkins AH (1999). Pathography: Patient Narratives of Illness. The Western Journal of Medicine.

[CR29] ———. 1999b. *Reconstructing Illness: Studies in Pathography*. vol. 393. Lafayette, Indiana, US: Purdue University Press.

[CR30] Huang C-D, Liao K-C, Chung F-T, Tseng H-M, Fang J-T, Lii S-C, Kuo H-P, Yeh S-J, Lee S-T (2017). Different Perceptions of Narrative Medicine between Western and Chinese Medicine Students. BMC Medical Education.

[CR31] Kasparian NA, Mireskandari S, Butow PN, Dieng M, Cust AE, Meiser B, Barlow-Stewart K, Menzies S, Mann GJ (2016). ‘Melanoma: Questions and Answers.’ Development and Evaluation of a Psycho-educational Resource for People with a History of Melanoma. Supportive Care in Cancer.

[CR32] Kleinman A (1988). *The Illness Narratives: Suffering, Healing, and the Human Condition*. New York City.

[CR33] Lamprell K, Braithwaite J (2016). Patients as Story-tellers of Healthcare Journeys. Medical Humanities.

[CR34] ———. 2017. When Patients tell Their own Stories: A Meta-narrative Story of Web-based Personalized Texts of 214 Melanoma Patients’ Journeys in Four Countries. *Qualitative Health Research*. 10.1177/1049732317742623. 10.1177/1049732317742623 29173015

[CR35] Lee EH, Klassen AF, Lawson JL, Cano SJ, Scott AM, Pusic AL (2016). Patient Experiences and Outcomes following Facial Skin Cancer Surgery: A Qualitative Study. The Australasian Journal of Dermatology.

[CR36] Lejeune P, Tomarken A, Tomarken E (1977). Autobiography in the Third Person. New Literary History.

[CR37] Lopez KA, Willis DG (2004). Descriptive versus Interpretive Phenomenology: Their Contributions to Nursing Knowledge. Qualitative Health Research.

[CR38] Mattingly C (1998). *Healing Dramas and Clinical Plots: The Narrative Structure of Experience*.

[CR39] Mattingly C, Garro LC (2000). *Narrative and the Cultural Construction of Illness and Healing*.

[CR40] Mattingly C, Lawlor M (2000). Learning from Stories: Narrative Interviewing in Cross-cultural Research. Scandinavian Journal of Occupational Therapy.

[CR41] Melanoma Institute Australia. 2017. “What is Melanoma?” Accessed 1 May 2017. https://www.melanoma.org.au/understanding-melanoma/what-is-melanoma/.

[CR42] Miller E, Balmer D, Hermann N, Graham G, Charon R (2014). Sounding Narrative Medicine: Studying Students' Professional Identity Development at Columbia University College of Physicians and Surgeons. Academic Medicine.

[CR43] Mishler EG (1984). *The Discourse of Medicine: Dialectics of Medical Interviews*. Norwood.

[CR44] O’Brien MR, Clark D (2010). Use of Unsolicited First-person Written Illness Narratives in Research: Systematic Review. Journal of Advanced Nursing.

[CR45] ———. 2012. Unsolicited Written Narratives as a Methodological Genre in Terminal Illness: Challenges and Limitations. *Qualitative Health Research* 22 (2): 274-284. 10.1177/1049732311420737 21876209

[CR46] Oliveria Susan A., Shuk Elyse, Hay Jennifer L., Heneghan Maureen, Goulart Jacqueline M., Panageas Katherine, Geller Alan C., Halpern Allan C. (2011). Melanoma survivors: health behaviors, surveillance, psychosocial factors, and family concerns. Psycho-Oncology.

[CR47] Oyebode F (2010). The Medical Humanities: Literature and Medicine. Clinical Medicine (London, England).

[CR48] Palesh O, Aldridge-Gerry A, Bugos K, Pickham D, Chen JJ, Greco R, Swetter SM (2014). Health Behaviors and Needs of Melanoma Survivors. Supportive Care in Cancer.

[CR49] Rebecca VW, Sondak VK, Smalley KSM (2012). A Brief History of Melanoma: from Mummies to Mutations. Melanoma Research.

[CR50] Riessman CK, Gubrium JF, Holstein JA (2002). Analysis of Personal Narratives. *Handbook of Interview Research: Context and Method*.

[CR51] Rosti G (2017). Role of Narrative-based Medicine in Proper Patient Assessment. Supportive Care in Cancer.

[CR52] Stamataki Z, Brunton L, Lorigan P, Green AC, Newton-Bishop J, Molassiotis A (2015). Assessing the Impact of Diagnosis and the Related Supportive Care Needs in Patients with Cutaneous Melanoma. Supportive Care in Cancer.

[CR53] Viney W, Callard F, Woods A (2015). Critical Medical Humanities: Embracing Entanglement, Taking Risks. Medical Humanities.

